# C-851-07. Reassessing Gabapentin: Early Postoperative Use and Complications After Split-Thickness Skin Grafting in Burn Patients

**DOI:** 10.1093/jbcr/irag033.059

**Published:** 2026-04-06

**Authors:** Matthew Q Dao, Arman M Chowdhury, Lorden Sarah Hoff, Paul Won, BaiJing Qin, Amina El Ayadi, Jong Lee, Steven E Wolf

**Affiliations:** University of Texas Medical Branch - John Sealy School of Medicine, Galveston, TX; University of Texas Medical Branch - John Sealy School of Medicine, Galveston, TX; University of Texas Medical Branch - John Sealy School of Medicine, Galveston, TX; Division of Plastic Surgery, Department of Surgery, McGovern Medical School, University of Texas Health Science Center at Houston, Houston, TX; Division of Plastic Surgery, Department of Surgery, McGovern Medical School, University of Texas Health Science Center at Houston, Houston, TX; Division of Burn and Trauma Surgery, Department of Surgery, University of Texas Medical Branch, Galveston, Galveston, TX; Division of Burn and Trauma Surgery, Department of Surgery, University of Texas Medical Branch, Galveston, Galveston, TX; Division of Burn and Trauma Surgery, Department of Surgery, University of Texas Medical Branch, Galveston, Galveston, TX

## Abstract

**Introduction:**

Gabapentin is widely prescribed for neuropathic pain in burn patients, yet its impact on surgical recovery after split-thickness skin grafting (STSG) remains unclear. While this medication may provide analgesic benefits, concerns arise regarding its potential effects on wound healing. This study aimed to determine whether gabapentin use after STSG is associated with adverse postoperative outcomes.

**Methods:**

A retrospective cohort analysis was conducted using the Global Collaborative Network within TriNetX, a multi-institutional database of electronic health records. Burn patients over the age of 18 years who underwent STSG between the years of 2010 and 2025 were identified. Two cohorts were established: those prescribed gabapentin (300–800 mg) within 1 week postoperatively (n = 5017) and those without any prescription of gabapentin following surgery (n = 17 421). Propensity score matching was then employed with a 1:1 ratio for demographics (sex, age, ethnicity, race), co-morbidities (chronic obstructive pulmonary disease, cerebrovascular diseases, obesity, congestive heart failure, liver disease, diabetes, hypertension, and chronic kidney disease), body mass index, site of bodily injury, degree of burn, and percentage of total body surface area affected. Primary outcomes included surgical site infection, cellulitis, blood transfusion, emergency department (ED) utilization, and graft failure at 30 days after STSG. Secondary outcomes evaluated hypertrophic scar formation and contractures, which were assessed at 90 days and 1 year postoperatively. Risk ratios (RR) were calculated for each outcome, with statistical significance set as *p*<.05.

**Results:**

Following matched analysis, each cohort had 4843 patients. At 30 days following surgery, matched patients who were prescribed gabapentin within one week of STSG had significantly higher rate of surgical site infection (RR 1.65, *p*=.039), cellulitis (RR 1.53, *p*=.010), need for transfusion (RR 1.43, *p*=.005), utilization of ED services (RR 2.22, *p*<.0001), and graft failure (RR 1.51, *p*=.013) compared to the matched patients who did not receive gabapentin. Furthermore, rates of contractures were significantly greater in the gabapentin cohort versus the control cohort at 90 days (RR 1.95, *p*<.0001) and 1 year (RR 1.66, *p*<.0001) of STSG. However, no significant differences were observed for hypertrophic scar formation between the two matched cohorts.

**Conclusions:**

Gabapentin use early after STSG was linked to higher rates of infection, graft failure, and contracture in burn patients. These results indicate the need to reconsider the prescription of this medication after recent surgery and encourage further investigation on its effects in burn care.

**Applicability of Research to Practice:**

Surgeons should exercise caution when prescribing gabapentin immediately after STSG and consider safer alternative pain control strategies to better facilitate wound healing and recovery.

**Funding for the Study:**

N/A.

